# Sleep architecture based on sleep depth and propensity: patterns in different demographics and sleep disorders and association with health outcomes

**DOI:** 10.1093/sleep/zsac059

**Published:** 2022-03-10

**Authors:** Magdy Younes, Bethany Gerardy, Allan I Pack, Samuel T Kuna, Cecilia Castro-Diehl, Susan Redline

**Affiliations:** Sleep Disorders Centre, University of Manitoba, Winnipeg, Manitoba, Canada; YRT Ltd., Winnipeg, Manitoba, Canada; YRT Ltd., Winnipeg, Manitoba, Canada; Division of Sleep Medicine/Department of Medicine, University of Pennsylvania, Perelman School of Medicine, Philadelphia, PA, USA; Division of Sleep Medicine/Department of Medicine, University of Pennsylvania, Perelman School of Medicine, Philadelphia, PA, USA; Department of Medicine, Corporal Michael J. Crescenz Veterans Affairs Medical Center, Philadelphia, PA, USA; Department of Medicine, Division of Sleep and Circadian Disorders, Brigham and Women’s Hospital, Harvard Medical School, Boston, MA, USA; Department of Medicine, Division of Sleep and Circadian Disorders, Brigham and Women’s Hospital, Harvard Medical School, Boston, MA, USA

**Keywords:** odds ratio product, ORP, sleep architecture, quality of life, Epworth sleepiness scale, obstructive sleep apnea, insomnia

## Abstract

**Study Objectives:**

Conventional metrics of sleep quantity/depth have serious shortcomings. Odds-Ratio-Product (ORP) is a continuous metric of sleep depth ranging from 0 (very deep sleep) to 2.5 (full-wakefulness). We describe an ORP-based approach that provides information on sleep disorders not apparent from traditional metrics.

**Methods:**

We analyzed records from the Sleep-Heart-Health-Study and a study of performance deficit following sleep deprivation. ORP of all 30-second epochs in each PSG and percent of epochs in each decile of ORPs range were calculated. Percentage of epochs in deep sleep (ORP < 0.50) and in full-wakefulness (ORP > 2.25) were each assigned a rank, 1–3, representing first and second digits, respectively, of nine distinct types (“1,1”, “1,2” … ”3,3”). Prevalence of each type in clinical groups and their associations with demographics, sleepiness (Epworth-Sleepiness-Scale, ESS) and quality of life (QOL; Short-Form-Health-Survey-36) were determined.

**Results:**

Three types (“1,1”, “1,2”, “1,3”) were prevalent in OSA and were associated with reduced QOL. Two (“1,3” and “2,3”) were prevalent in insomnia with short-sleep-duration (insomnia-SSD), but only “1,3” was associated with poor sleep depth and reduced QOL, suggesting two phenotypes in insomnia-SSD. ESS was high in types “1,1” and “1,2”, and low in “1,3” and “2,3”. Prevalence of some types increased with age while in others it decreased. Other types were either rare (“1,1” and “3,3”) or high (“2,2”) at all ages.

**Conclusions:**

The proposed ORP histogram offers specific and unique information on the underlying neurophysiological characteristics of sleep disorders not captured by routine metrics, with potential of advancing diagnosis and management of these disorders.

**Statement of Significance**

Interpretation of the electroencephalogram obtained in sleep studies continues to follow guidelines introduced by Rechtschaffen and Kales (R&K) in the 1960s. These guidelines provide very limited information on sleep depth and sleep propensity, which vary over a wide range within the conventional R&K sleep stages. The odds ratio product (ORP) was recently introduced and validated as a continuous measure of sleep depth. In this report, we introduce a new way of looking at sleep using ORP. Here, sleep architecture is described as percent of recording time spent in each of ten deciles within the total ORP range. This approach has resulted in unique patterns that shed new light on the pathophysiology underlying various sleep disorders.

## Introduction

Assessment of sleep depth and duration by clinical polysomnography (PSG) provides information on the neurophysiological characteristics of sleep disorder subtypes, which may reflect pathophysiologic mechanisms and influence symptoms and outcomes of the sleep disorder. At present the neurophysiology of sleep is most commonly assessed from total sleep time (TST), sleep efficiency (SE), and percent of time in stages N1 and N3. Frequency of arousals and awakenings is also commonly utilized as a measure of sleep continuity. These metrics have had limited diagnostic or prognostic value such that PSG is no longer indicated for investigation of a highly prevalent sleep disorder, insomnia [1], and resulted in a major shift from PSG to home testing for another very common sleep disorder, obstructive sleep apnea (OSA).

The scoring rules for sleep stages and arousals were developed decades ago [2, 3] with only minor modifications since, and they were designed for visual implementation. Because of the limitations of visual scoring, rules were based on features that could easily be identified and estimated visually. Technological advances since their introduction makes it clear that these rules are seriously limited. The limitations include (See Supplementary Material for more details): (a) the omission or failure to quantify differences in sleep propensity during stage wake, and variations in sleep depth within each sleep stage [4, 5], (b) inter-rater variability in scoring stages [6], (c) inappropriate reliance on spindles and slow waves to indicate deeper sleep (relative to N1 and N2, respectively) [7], (d) lack of consistency in responses of conventional metrics [8], and (e) inability of the arousal index to consider the variable duration [9] or intensity [10] of arousals, potential adaptation of arousal threshold over time, or the variable after-effects of arousals on sleep depth [11].

The odds ratio product (ORP) is a continuous index of sleep depth and propensity, ranging from 0 (very deep sleep) to 2.5 (full wakefulness) [4]. Much evidence supports ORP in this regard [4, 7, 8, 12, 13] including a high correlation (*r*^2^ = 0.98) between ORP in any given epoch and probability of arousal/awakening occurring in the next 30 seconds [4, 7].

**Figure 1.**
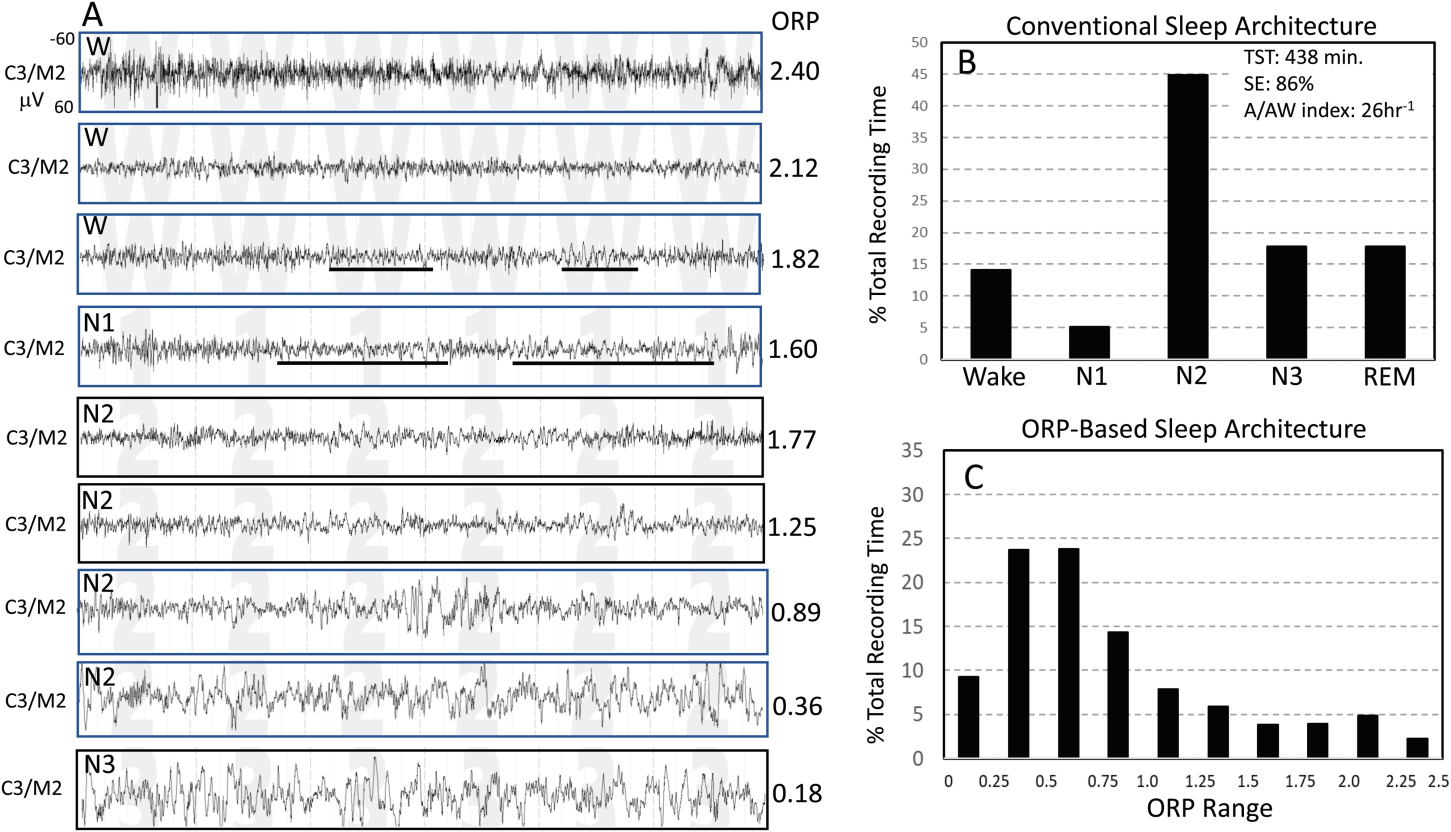
Records from one subject from the Sleep Heart Health Study. (A) Nine 30-second EEG tracings representing EEG patterns receiving ORP values spanning the entire ORP range (0.00–2.50). Epochs with ORP > 1.75 are typically scored wake but exhibit a wide spectrum from full wakefulness with high ORP (top panel) to patterns with sleep features (theta activity and micro-sleep) but do not meet the criteria of sleep. Likewise, a wide range of patterns can be identified in epochs typically scored NREM sleep. The figure shows ORP values ranging 0.36–1.77 within stage N2. (B) Conventional sleep stages in the same subject showing normal values. (C) The proposed ORP-based architecture in which % of epochs occurring within each ORP decile is illustrated. Deciles 1 and 2 represent very deep and deep sleep, respectively (cf. A, two bottom epochs). Decile 3 is moderate sleep and decile 4 is light sleep. Deciles 5–7 are transitional states with progressively increasing wake features (alpha-beta rhythms). Deciles 8 and 9 represent epochs typically scored wake but with sleep features. Decile 10 is seen in full wakefulness (A, top panel).

Within a given individual, ORP decreases progressively as state progresses from full wakefulness to deep sleep (Figure 1A) [4, 8, 14]. However, there are considerable interindividual differences in average ORP in different stages depending on the distribution of epochs with different ORP values within the same stage [4, 15]. Because ORP is a continuous metric, time spent in different ORP ranges can be calculated, providing more precise and comprehensive information about sleep depth and wake propensity.

In this communication, we introduce an ORP-based approach that avoids the limitations of conventional scoring. We describe how the patterns so identified vary with individual characteristics and sleep disorders in a large community-based cohort. We hypothesized that such information would lead to better understanding of the impact of sleep disorders on sleep. Our intent is not to replace the familiar and long serving conventional evaluation. Rather, we hope that the proposed approach may supplement the current approach by providing clinically useful insights not currently evident using the Rechtschaffen’ and Kales’ approach.

## Methods

We used two pre-existing datasets: an observational study, the “Sleep Heart Health Study (SHHS1)” [16] and an experimental study that evaluated effects of acute sleep deprivation on a sample of monozygotic and dizygotic twins [17].

SHHS1 [16] is a community-based study of adults 40–90 years old. SHHS findings are available through the National Sleep Research Resource (NSRR; sleepdata.org) [18]. For this study, we obtained the PSGs (available in 5804 subjects), demographics, conventional PSG scoring and questionnaires documenting insomnia symptoms, sleepiness (Epworth Sleepiness Scale; ESS), and quality of life (Short-Form-Health-Survey-36; SF36, and its physical and mental components SF-36-P and SF-36-M, respectively). SHHS data were used earlier to obtain normative values of ORP and other EEG biomarkers [5]. Here, ORP data generated earlier [5] were further analyzed to obtain ORP-based architecture.

Clinical categories were identified from questionnaires and manually scored data. Participants with total recording time <7 h (2197) were not included to exclude influences of short battery life on the data and to facilitate analysis of insomnia with short sleep duration (insomnia-SSD) [19–22]. As a result, data from 3585 subjects were used in the current study. OSA was categorized as mild (AHI 5–15/h), moderate (AHI 15–30/h), severe (AHI 30–50/h), or very severe (>50/h). AHI was based on apnea events plus hypopneas with ≥30% amplitude reduction plus 4% decrease in oxygen saturation. Presence of insomnia was based on a report of difficulty falling asleep, waking up too early or waking up and having difficulty resuming sleep, if any of these symptoms occurred >16 times per month (scored 5 on the insomnia questionnaire’s 1–5 scale). “Insomnia + OSA” (COMISA) was designated if participants met criteria of insomnia and had an AHI >5/h. Those with insomnia without OSA were further divided into those with short sleep duration (TST < 6 h, insomnia-SSD) or with normal sleep duration (TST ≥ 6 h, insomnia-NSD). Subjects with neither insomnia nor OSA were classified as “No OSA/Insomnia”.

The experimental study [17] examined heritability of response to acute sleep deprivation in 100 twin pairs (59 monozygotic and 41 dizygotic pairs). OSA-free participants, 18–53 years old, underwent baseline PSG, followed by 36 h of sleep deprivation and a recovery PSG. This study was previously scored to determine changes in ORP after sleep deprivation [12]. Here, they were used to extract the new ORP-metrics in subjects aged 18–39 (*n* = 171), as this age group was not available in SHHS1, and to document the effect of sleep deprivation on ORP metrics.

### Analytical methods

The method of measuring ORP was described previously [4]. A brief description is provided in the Supplement. ORP is measured in 3-second epochs. For this study average of the ten 3-second epochs within each 30-second epoch will be reported.

**Figure 2.**
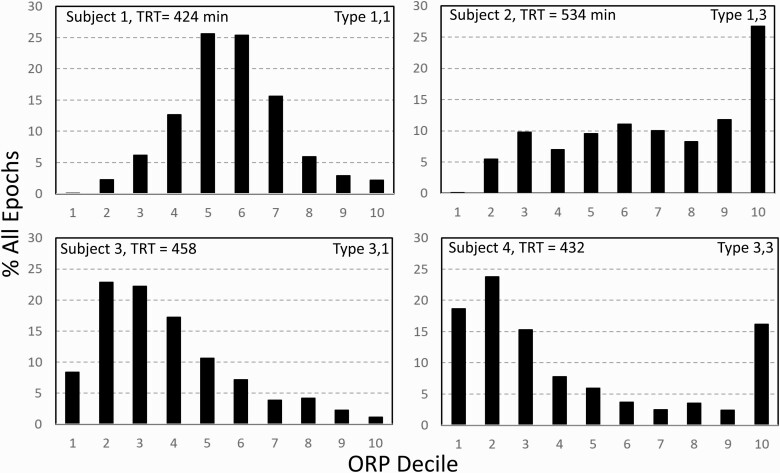
Four architecture patterns randomly found in SHHS participants showing different relations between % of epochs in deep sleep (deciles 1 and 2) and full wakefulness. Participant 1, both ends of the spectrum are low. Participant 2, deep sleep is low while decile 10 is high. Participant 3, much deep sleep with very little full wakefulness. Participant 4, many epochs both in deep sleep and full wakefulness. TRT, total recording time.

The ORP range (0.0–2.5) was divided into deciles and the percent of all 30-second epochs in the PSG within each ORP decile was calculated. This frequency distribution varied substantially among participants (e.g., Figure 2). Thus, fractions in deep sleep (deciles 1 and 2), and in full wakefulness (decile 10), varied in different ways, from both being relatively low (subject 1) or high (subject 4), to deep sleep being high with little full wakefulness (subject 2), and vice versa (subject 3). We consider that these different patterns reflect different sleep pathophysiology.

The rationale for the classification we used is as follows: High amounts of full wakefulness is interpreted as having low sleep pressure over a large fraction of the study, while very low amounts of full wakefulness across the sleep period suggests that sleep was not fully restorative. Thus, high proportions of full wakefulness with low levels of deep sleep (subject 2, Figure 2) would suggest low sleep pressure throughout (e.g. hyperarousal state), while high proportions of full wakefulness with high amounts of deep sleep (subject 4, Figure 2) would suggest circadian misalignment (e.g. a subject with delayed sleep phase disorder starting the study at 9 pm) or excessive time in bed. Likewise little deep sleep with little time in full wakefulness (subject 1, Figure 2) would suggest a process that interferes with progression to deep sleep (e.g. OSA, other stimuli) and simultaneously results in high sleep pressure while abundant deep sleep with little full wakefulness (subject 3, Figure 2) would suggest inadequate time in bed, high sleep need, or prior sleep deprivation.

Each participant was assigned a 2-digit type based on the distribution of values in the different deciles among the entire cohort (*n* = 3585). Sum of percent of epochs in deciles 1 and 2 (ORP < 0.5) was considered as % of epochs in deep sleep. That percent averaged 20.1 ± 13.2%TRT (Range 0.0%–79.0%) with an interquartile range of 10.2%–28.5%TRT. The first digit was assigned a value of “1” if % epochs in deep sleep was in the first quartile (i.e. <10.2%), a value of “2” if % was in the interquartile range (10.2%–28.5%TRT), and a value of “3” if % of deep sleep was >28.5. Percent of epochs in full wakefulness (decile 10, ORP > 2.25) averaged 9.3 ± 8.4%TRT (Range 0.0%–70.1%) with an interquartile range of 3.4%–12.5%TRT. The second digit was assigned “1” if % of epochs in decile 10 was in the first quartile of its distribution (i.e. <3.4%), “2” if in the interquartile range (3.4%–12.5%) and “3” if >12.5%TRT. Thus, type “1,1”, indicates that both deep sleep and full wakefulness are in their respective lowest quartiles (Subject 1, Figure 2)…etc.

**Figure 3.**
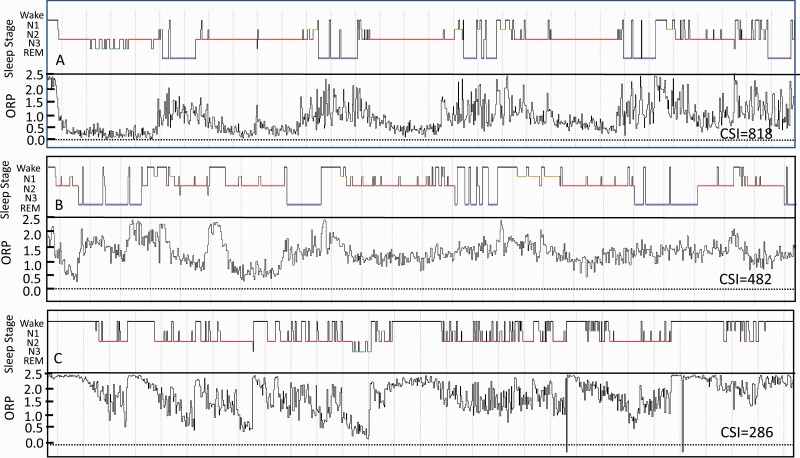
Compressed full night studies from three SHHS participants illustrating the Cumulative Sleep index (CSI, area between full wakefulness (ORP = 2.5) line and the epoch-by epoch ORP tracing). In practice this is calculated from [(2.50 – ORP in total recording time (TRT)) multiplied by TRT in minutes]. The corresponding conventional histograms are also shown. Note the marked difference in CSI between the three studies. ORP, odds ratio product; REM, rapid eye movement sleep; N1, N2, and N3 are stages 1–3 of non-REM sleep.

In addition, the cumulative reduction in ORP across the total study from full wakefulness was calculated for each participant from [(2.5-ORP_TRT_) * TRT], where ORP_TRT_ is average ORP in all epochs, and total recording time (TRT) is in minutes. This value is referred to as Cumulative Sleep Index (CSI; Figure 3) and is intended to provide a quantitative metric of the overall “wakefulness reduction” achieved during each study.

### Statistical analyses

Association of *age, gender and BMI* with ORP-architecture: Four age ranges were compared: One group consisted of subjects under 40 in the sleep deprivation study [17] (*n* = 171) while SHHS1 participants with “No OSA/Insomnia” and with TRT >7 h (*n* = 1517) were separated into three groups: 39–54, 55–69, and >70 years. ORP-architecture in these three groups was compared to that in the youngest group. For *gender association*, ORP-architecture in males and females of the “No OSA/Insomnia” participants were compared. For *associations with BMI*, “No OSA/Insomnia” subjects were divided into three BMI groups: 18 to <26, 26 to <30, and ≥30 kg/m^2^ and results of the two heavier groups were compared to the leanest group. For all comparisons we used the independent *t* test, where there were only two groups or one-way analysis of variance (ANOVA) for >two groups to compare group values at each ORP decile. If *p* < .05, each group within the same decile was compared to the first group using the independent *t*-test with appropriate Bonferroni correction.

Association of *clinical sleep phenotypes* with ORP-architecture: for each ORP decile we compared average percent of epochs (%TRT) in each of the four OSA severity levels with the percent of epochs in the same decile in the “No OSA/Insomnia” group using one-way ANOVA. The same was done for the three groups of Insomnia (insomnia-NSD, insomnia-SSD, COMISA), comparing percent of epochs in each group/decile with percent in the same decile in the “No OSA/Insomnia” group. If *p* < .05, each group within the same decile was compared to the first group using the independent t-test with appropriate Bonferroni correction. In addition, for each of the seven clinical groups we used the chi-square test to identify differences between the distribution of different ORP types in the clinical group from type distribution in the “No OSA/Insomnia” group.

The impact of *changes in sleep pressure* was assessed in two ways. Results before and after 36 h of sleep deprivation in the twin study [17] were compared using the paired t test to determine the changes in ORP architecture as sleep pressure increased. To determine the impact of reduced sleep pressure, each PSG from participants in SHHS classified as “No OSA/Insomnia” was divided into first and second halves after excluding sleep latency. ORP-architecture in the two sections was compared using the paired *t* test.

Average ORP in stages wake (ORP_W_) and all NREM sleep (ORP_NR_) and in TRT (ORP_TRT_) as well as CSI in each architecture type was calculated. In addition, because of its relevance to control of sleep depth [11], average ORP-9 (speed of sleep recovery following arousals) [11] was determined.

Finally, average ESS, SF36-P, and SF36-M were calculated for SHHS participants in each ORP-type and the averages across types were compared using one-way ANOVA. Differences in these metrics were also calculated after adjusting for differences in age, gender, BMI, AHI, and insomnia between ORP types.

## Results

Figure 1A illustrates the different levels of ORP that occur within stage Wake (top three tracings), stage N2 sleep (tracings 5–8), and stage N3 (bottom tracing) in a SHHS1 participant and compares the conventional architecture (Panel B) with the proposed ORP-architecture (Panel C). There was little difference in ORP between the last two tracings (0.36 vs. 0.18), reflecting the minimal difference in their visual appearance even though one was staged N2 and the other as N3. The differences in ORP among the three top wake epochs are also of note. Supplementary Figure S1 shows a striking example of wake epochs with low ORP in a SHHS subject with severe OSA where transient dips in ORP are followed by obstructive events with re-awakening within the same epoch.

### Sensitivity analysis

Many of the variables included in the following analyses were not normally distributed. However, sensitivity analyses removing highly influential points did not materially affect the results. Furthermore, use of nonparametric tests, Wilcoxon (instead of *t*-test) or Kruskal–Wallis (instead of ANOVA), in such cases did not alter the results (data not shown).

### Variation with demographics and BMI

**Figure 4.**
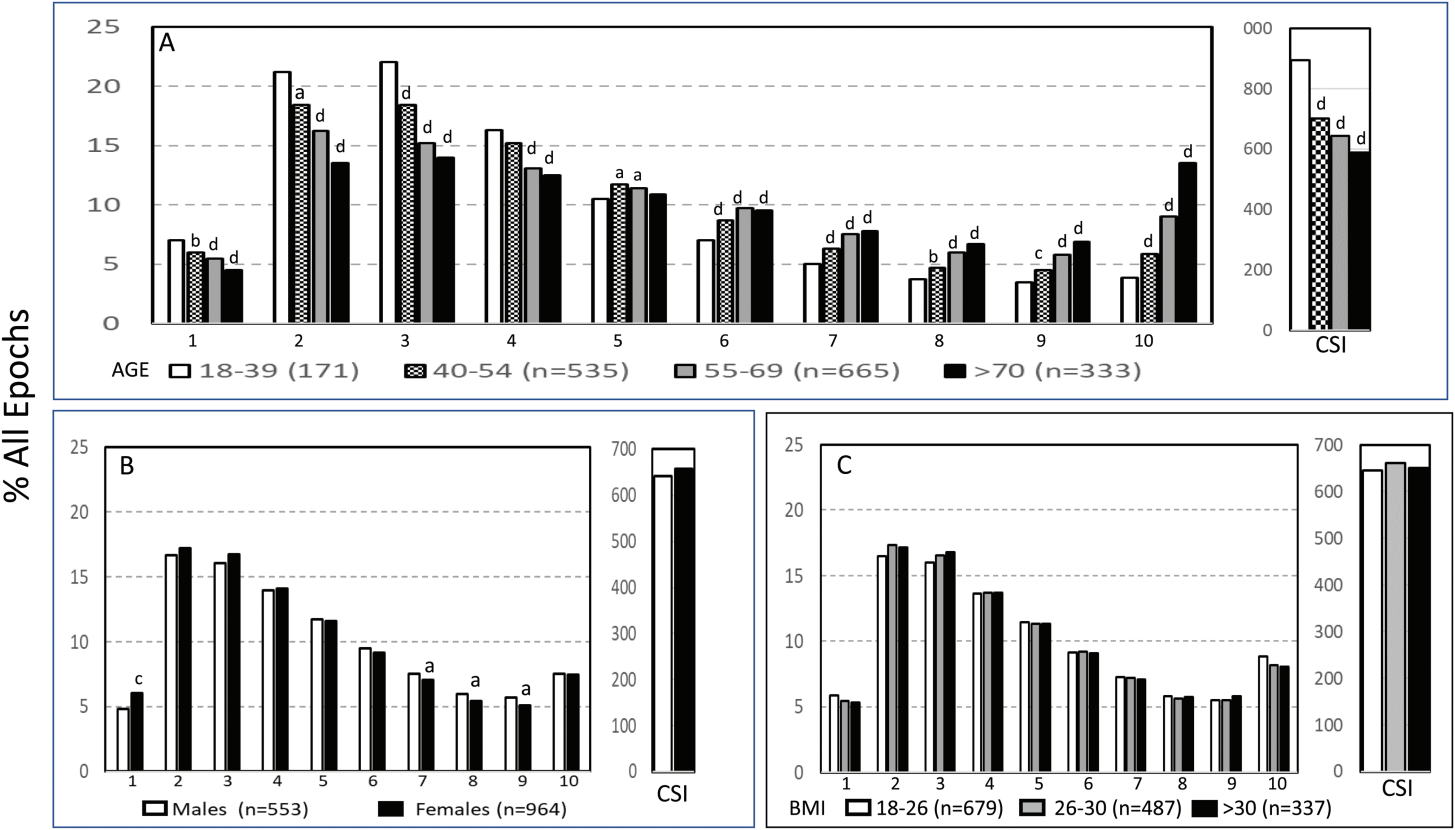
(A) Changes in ORP-architecture with age (A), gender (B), and body mass index (BMI, C) in participants with “No OSA/Insomnia” in the Sleep Heart Health Study. Abscissa values are the odds ratio product (ORP) deciles, with decile 1 representing the deepest sleep (0.00–0.25), and decile 10 representing full wakefulness (ORP > 2.25). The different groups at each decile were compared by one-way analysis of variance (ANOVA). If *p* < .05, each group within the same decile was compared to the first group (youngest in the case of age) using the independent *t*-test with appropriate Bonferroni correction. Significant differences from the first group are indicated by letters: “a”, *p* ≤ .05; “b”, *p* ≤ .01; “c”, *p* ≤ .001; “d”, *p* ≤ .0001. CSI, Cummulative Sleep Index.

Figure 4 shows the variation in ORP-architecture with age, gender, and BMI. Percent of epochs in the lowest four deciles (ORP < 1.0), which represent very deep (ORP < 0.25) to light sleep (0.75–1.00), progressively decreased, while percent of epochs in transitional and drowsy-wake deciles (deciles 5–9) increased progressively with age. The increase in the highest decile (ORP >2.25, full wakefulness) was particularly striking as percent of epochs increased threefold in the oldest age group by comparison to the youngest group. CSI decreased from 895 ± 196 to 589 ± 133 ORP units * min, a 34% decrease from the youngest (twin cohort) to the oldest (>70 years) age group (*p* < .0001) (Inset, Figure 4A).

Women had slightly more epochs in deep sleep than men and slightly fewer epochs in transitional and drowsy wake states (Figure 4B). CSI was marginally higher in women (*p* = .03; Inset Figure 4B). ORP-architecture was not associated with BMI (Figure 4C).

### Changes with sleep pressure

**Figure 5.**
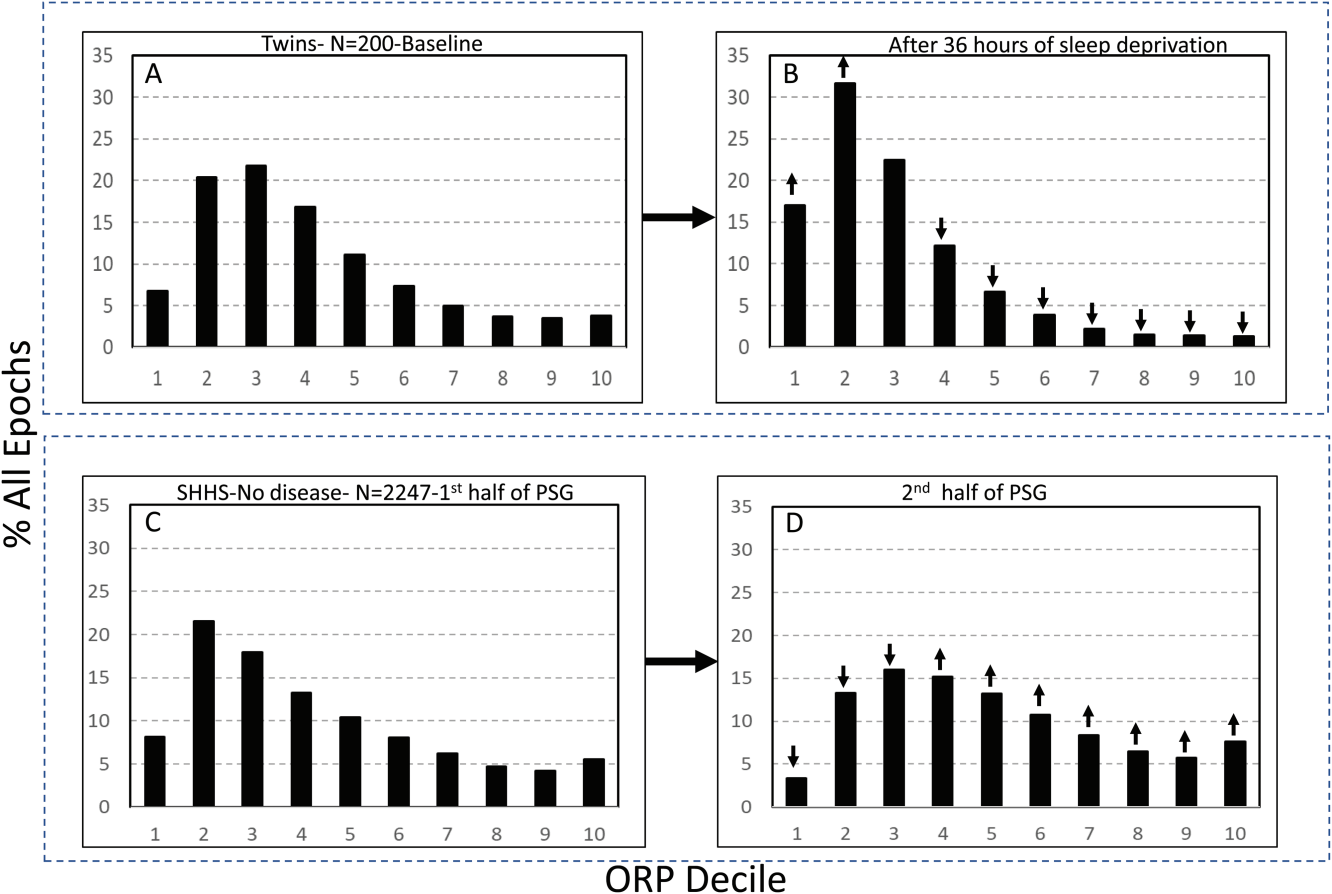
(A) and (B) Odds ratio product (ORP) architecture in 200 healthy participants in overnight polysomnograms before and following 36 h of sleep deprivation. Note the remarkable leftward shift in the distribution. (C) and (D) Comparison of ORP-architecture in the first and second halves of the night in Sleep Heart Health Study (SHHS) subjects with “No OSA/Insomnia”. An opposite shift is evident.↓ and↑, significant increase or decrease relative the same decile in the reference panel (*p* < 1.E−10). PSG, polysomnogram.

Figure 5 shows changes in ORP-architecture with increases (panels A and B) and decreases (panels C and D) in sleep pressure. Thirty-six hours of sleep deprivation was followed by remarkable leftward shift in the distribution with marked increases in deciles 1 and 2, decreases in transitional and wake states, and virtually no epochs in full wakefulness (Figure 5B; 1.3%). In contrast, decline in sleep pressure from the first to the second half of sleep period time resulted in a rightward shift in the histogram (Figure 5C and D).

### Variation in ORP architecture across clinical sleep phenotypes

**Figure 6.**
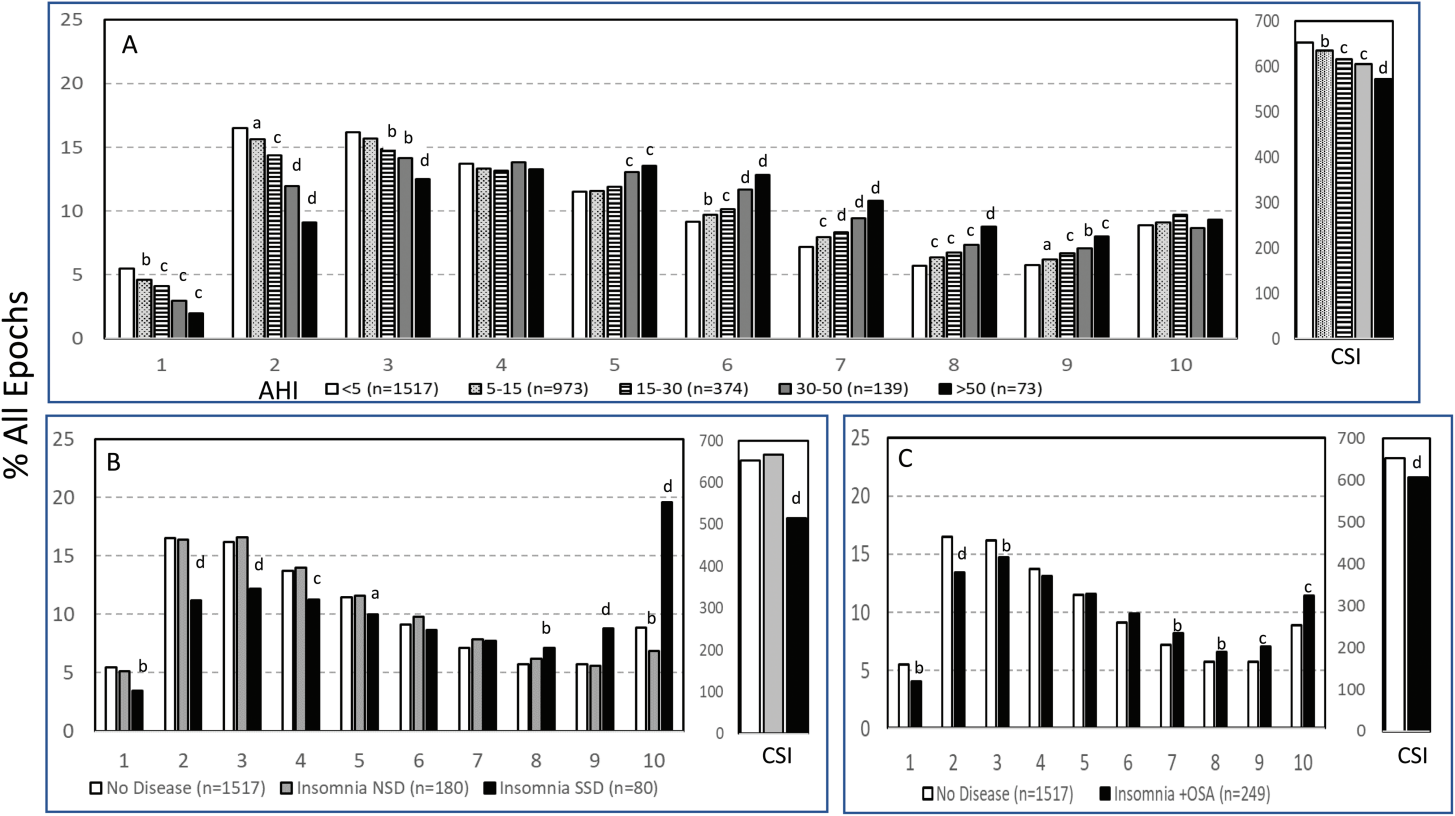
Changes in ORP-architecture (ORP = odds ratio product) with increasing obstructive apnea (OSA) severity (A), different types of insomnia (B), and in participants with insomnia plus OSA (C). AHI, apnea hypopnea index; NSD, normal sleep duration; SSD, short sleep duration. Abscissa values are the ORP deciles, with decile 1 representing the deepest sleep (0.00–0.25), and decile 10 representing full wakefulness (ORP > 2.25). The different groups at each decile were compared by one-way analysis of variance (ANOVA). If *p* < .05, each group within the same decile was compared to the first group using the independent *t*-test with appropriate Bonferroni correction. Significant differences from the first group are indicated by letters: “a”, *p* ≤ .05; “b”, *p* ≤ .01; “c”, *p* ≤ .001; “d”, *p* ≤ .0001.

Increasing OSA severity was associated with progressively fewer epochs in the lower deciles and progressively more in transitional sleep and drowsy wakefulness (Figure 6A). Although these associations were modestly related to age differences across OSA stratum, age-adjusted analyses showed that the percentage of epochs in decile 2 decreased from 16.1 ± 8.6% to 9.5 ± 8.3%, a difference of 6.6% (*p* < .0001), which is only marginally lower than the difference before age adjustment (7.4%; *p* < .0001).

Cumulative sleep index (CSI) decreased from 652 ± 132 in the “No OSA/Insomnia” group to 572 ± 124 ORP units * TRT in very severe OSA (*p* < .0001; Inset, Figure 6A). The change after age adjustment was 642 ± 125 to 581 ± 123 ORP units * TRT, also highly significant (*p* < .0001). The reduction in adjusted CSI corresponded to 2, 5, 7, and 9% of the “No OSA/Insomnia” value as OSA severity increased from mild to very severe.

There were no significant differences between groups defined as having insomnia-NSD and those with “No OSA/Insomnia” except for reduction in epochs in full wakefulness (6.9% vs. 8.9%; *p* < .001; Figure 6B), while the group classified as insomnia-SSD had fewer epochs in the low deciles and more epochs in the higher deciles. The increase in the highest decile (ORP > 2.25) in insomnia-SSD was particularly striking (Figure 6B). CSI was markedly reduced in this group (515 ± 103 vs. 652 ± 132 ORP units * TST; *p* < .0001). Differences between individuals with COMISA and “No OSA/Insomnia” were similar to, but milder than, in those with insomnia-SSD (Figure 6C).

### Association of % time in deep sleep (ORP < 0.5) and full wakefulness (ORP > 2.25) with self-reported sleepiness and quality of life

Of relevance to the main rationale of this study, in multiple regression analysis including age, gender and BMI, ESS was inversely associated with time spent in full wakefulness (estimate −0.035, *p* = .0002; Table 1). Time in full wakefulness also had a negative association with SF36-P (Table 1). While not associated with ESS, time in deep sleep (ORP < 0.5) had a strong positive association with SF-36-M (*p* < .0001).

**Table 1:** Association of sleepiness and quality of life with age, gender, BMI and % time in deep sleep (ORP < 0.5) and full wakefulness (ORP > 2.25)*

Variable	ESS (n = 3447)			SF36-P (n = 3270)			SF36-M (*n* = 3270)		
	Estimate	*t*-value	Pr > [*t*]	Estimate	*t*-value	Pr > [*t*]	Estimate	*t*-value	Pr > [*t*]
Overall Model	*r* ^2^ = 0.03, *p* < .0001			*r* ^2^ = 0.14, *p* < .0001			*r* ^2^ = 0.03, *p* < .0001		
Intercept	5.0	7.6	<0.0001	75.8	53.7	< 0.0001	45.1	35.1	<0.0001
Age (years)	−0.001	−0.1	0.92	−0.23	−16.0	**<0.0001**	0.11	7.9	**< 0.0001**
Female	−0.962	−6.5	**<0.0001**	−1.77	−5.6	**<0.0001**	−0.93	−3.2	**<0.001**
BMI (kg/m^2^)	0.091	6.1	**<0.0001**	−0.49	−15.3	**<0.0001**	−0.01	−0.31	0.76
Deep sleep (%TRT)	0.000	0.0	0.996	0.01	0.9	0.395	0.07	5.5	**<0.0001**
Full wakefulness (%TRT)	−0.035	−3.7	**0.0002**	−0.06	−3.0	**0.003**	−0.01	−0.3	0.77

* All eligible SHHS participants were included in the models. ORP, odds ratio product; ESS, Epworth Sleepiness Score; SF36(P), standardized score of SF-36 physical component; SF36-M, standardized score of SF-36 mental component. BMI, body mass index; TRT, total recording time. All three multiple linear regression models included all five variables, and each overall model was highly significant (*p* < .0001).

Bold values are individual variables that were significantly associated with the indicated health outcome by multiple linear regression.

### Conventional sleep metrics in the current cohort

Table 2 shows the distributions of conventional sleep metrics in the different clinical phenotypes. TST was, by definition, lower in insomnia-SSD than in insomnia-NSD. However, except for a slightly higher N1 sleep (6.4 (1.8–13.1) %TST vs. 4.6 (0.4–1.0) %TST; *p* < .0001), there were no differences in sleep stages (%TST) or in the arousal/awakening index between the two insomnia types or between either type and the “No OSA/Insomnia” group (Table 2). TST, SE, N3, and REM times were significantly lower than the No OSA/Insomnia group at all levels of AHI while N1 time was higher (Table 2).

**Table 2.** Conventional sleep variables in different clinical groups

PSG Variable	Sleep Heart Health Study 1 (*n* = 3585)								
	No OSA or Insomnia	Obstructive sleep apnea				Insomnia			ANOVA *p*=
		Mild	Moderate	Severe	Very severe	NSD	SSD	+OSA	
TRT (min)	468 (425–520)	469 (424–523)	468 (425–520)	471 (428–524)	473 (426–526)	468 (425–518)	470 (423–525)	471 (424–526)	.68
TST (min)	392 (306–462)	383* (292–456)	374* (269–451)	367* (270–443)	370* (271–451)	411* (364–463)	325* (256–392)	367* (257–452)	<.0001
SE (%)	83.9 (65.5–95.1)	81.9* (61.7–94.1)	79.* (57.0–93.8)	78.1* (57.2–92.4)	78.3* (58.8–93.5)	87.8* (80.6–94.6)	69.3* (54.4–79.4)	77.9* (54.5–93.6)	<.0001
N1 (%TST)	5.1 (0.8–11.4)	5.9* (1.0–13.0)	6.5* (0.9–15.3)	6.8* (0.5–15.5)	6.9* (0.9–18.8)	4.6 (0.4–10.0)	6.4* (1.8–13.1)	6.4* (0.7–17.1)	<.0001
N2 (%TST)	57.1 (38.7–75.5)	58.3 (39.9–76.0)	61.3* (43.9–80.0)	65.3* (47.7–87.7)	68.2* (50.3–89.7)	56.9 (40.2–76.9)	58.1 (35.9–81.0)	58.7 (39.7–78.7)	<.0001
N3 (%TST)	17.1 (0.7–35.4)	15.9* (0.2–36.5)	13.5* (0.2–31.5)	11.4* (0.0–31.7)	10.3* (0.0–30.3)	17.9 (0.0–33.6)	16.3 (0.0–35.4)	16.1 (0.0–34.7)	<.0001
REM (%TST)	20.7 (9.4–30.5)	19.9* (9.6–29.2)	18.7* (9.9–27.7)	16.5* (1.7–25.9)	14.7* (3.3–24.9)	20.6 (8.9–30.3)	19.2 (4.4–29.5)	18.8* (6.1–29.2)	<.0001
A/Aw index	23.1 (12.5–36.3)	27.1* (15.1–42.1)	31.6* (18.5–47.2)	37.7* (20.0–58.1)	48.4* (25.3–77.5)	23.6 (12.2–36.7)	24.4 (13.0–37.0)	29.3* (15.0–50.8)	<.0001
AHI (hr^−^1)	2.2(0.3–4.6)	8.8* (5.3–14.1)	21.0* (15.4–28.8)	38.1* (30.4–47.9)	63.2* (50.3–83.9)	2.1 (0.2–4.8)	2.3 (0.0–4.7)	17.0* (5.6–47.0)	<.0001
Total number	1517	973	374	139	73	180	80	249	3585

Values are Mean (5th–95th percentile). TRT, total recording time; TST, total sleep time; SE, sleep efficiency; REM, rapid eye movement sleep; NREM 1, 2, and 3, stages 1, 2, and 3 of non-REM sleep; A/Aw index, arousal/awakening index per hour; AHI, apnea hypopnea index; OSA, obstructive sleep apnea; NSD, insomnia with normal sleep duration; SSD, insomnia with short sleep duration. *, significantly different from the group with no OSA or insomnia by *t*-test at *p* < .007. Rectangle highlights the lack of difference in the lower five variables between the two insomnia groups and the “no OSA or Insomnia” group. Furthermore, there were no significant differences between the two insomnia groups in these five variables.

Of note, while the average differences from the “No OSA/Insomnia” group reported in Table 2 were highly significant, they represented small fractions of the ranges of these metrics, and there was almost complete overlap of the 5th–95th percentile range in any variable/clinical group and the corresponding range in the No OSA/Insomnia group.

### ORP-architecture types

**Figure 7.**
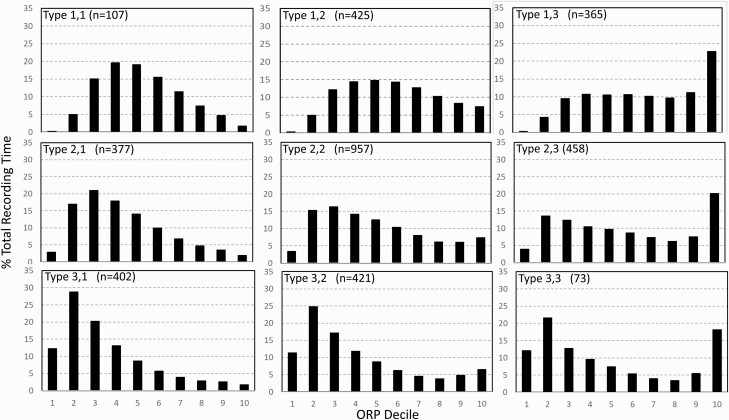
Average ORP-architecture in the nine pre-selected types. Number of subjects ranged 73–957 in the different types. The two numbers in the Type designation indicate the quartiles in which % of epochs in deep sleep (ORP < 0.5) and full wakefulness (last decile), respectively, were located; 1 = lowest quartile; 2 = interquartile range; 3 = highest quartile. ORP, odds ratio product. Standard error of the mean (not shown) was <0.7% for all columns in all nine patterns.

Figure 7 shows average architecture for all SHHS participants in each of the nine ORP architecture types. Fraction of epochs in deep sleep (deciles 1 and 2) increases from top to bottom while fraction in full wakefulness increases from left to right.

**Figure 8.**
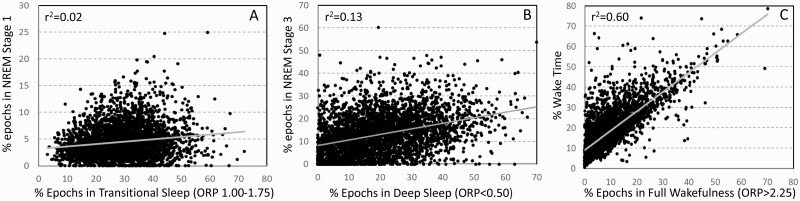
(A) Scatter plot of the relation between % epochs in transitional sleep (odds ratio product (ORP) 1.00–1.75) and % of epochs in stage N1 of NREM sleep. (B) Scatter plot of the relation between % epochs with ORP < 0.5 (deepest sleep) and in stage N3. (C) Scatter plot of the relation between % epochs in full wakefulness (ORP > 2.25) and % wake time.

ORP values corresponding to deep sleep (deciles 1 and 2), transitional sleep (deciles 5–7), and full wakefulness (decile 10) did not correspond to conventional metrics that evaluate the corresponding sleep attributes. Figure 8A shows no agreement (*r*^2^ = 0.02) between percent of epochs defined as transitional sleep by ORP and percent of epochs conventionally staged as N1. Likewise, percent of epochs designated as deep sleep by ORP (ORP < 0.5) shows only marginal agreement with percent of epochs in stage N3 (Figure 8B; *r*^2^ = 0.13). Amount in full wakefulness was better correlated with wake time (*r*^2^ = 0.60) but there was considerable scatter, particularly at low values of full wakefulness (Figure 8C). For example, at 5% full wakefulness, conventional wake time ranged from ≈5% to ≈30% TRT. The difference between conventional wake time and time in full wakefulness is a measure of time spent in drowsy wakefulness (ORP 1.75–2.25; Figure 1A). Thus, Figure 8C shows that a large fraction of time in stage wake may represent much time spent fully awake, or in a drowsy state, when the two conditions may reflect different underlying physiopathology (panel A vs. panel B, Supplementary Figure S1).

**Figure 9.**
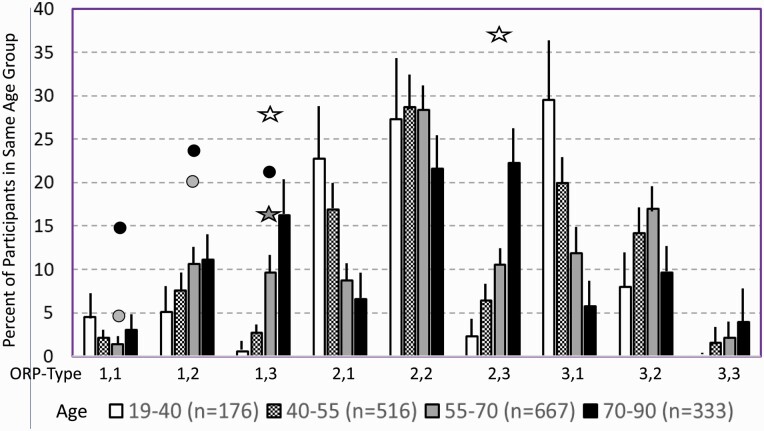
Prevalence of different ORP types in different age groups of participants with “No OSA/Insomnia” in both cohorts (Twins and Sleep Heart Health Study). Lines are upper margin of error (95% confidence interval). Solid circles, values found in participants with severe (grey circle), and very severe OSA (black circles) in the different ORP types (From Table 3). White stars, values found in participants with insomnia and short sleep duration (From Table 3). Dark stars, values found in participants with insomnia plus OSA (from Table 3). All symbols are plotted against the 55–70 age group (grey columns) since average age in all clinical groups fell in this range. Where no symbols are shown above a given ORP type, the prevalence of the type is within the confidence interval of participants with no OSA or insomnia.

Considering the strong association between age and % of epochs in different ORP deciles (Figure 4) we determined prevalence of ORP-types at different ages (Figure 9). In participants with “No OSA/Insomnia” type “2,2” was the dominant type in all age groups. Types “1,1” and “3,3” were rare in all age groups. The number of subjects with “1,2”, “1,3”, and “2,3” types increased with age while types “2,1” and “3,1” decreased with age. The same analysis was performed separately for males and females and there were no significant differences in ORP pattern distribution between the two genders within any age group or among the total participants in the two genders (Supplementary Table S1).

**Table 3:** Distribution of different ORP architecture types in clinical categories

ORP type	Sleep Heart Health Study								
	“No OSA/Insomnia”	Obstructive Sleep Apnea				Insomnia			All
		Mild	Mod.^a^	Sev.^b^	V. sev.^d^	NSD^c^	SSD^e^	+OSA^c^	
1,1	30 (2.0)	30 (3.1)	13 (3.5)	7^a^ (5.0)	11^d^ (15.1)	6 (3.3)	0 (0)	10 (4)	107
1,2	147 (9.7)	118 (12.1)	53 (14.2)	29^b^ (20.9)	18^b^ (24.7)	26 (14.4)	5 (6.3)	29 (11.6)	425
1,3	131 (8.6)	88 (9.0)	40 (10.7)	15 (10.8)	16^b^ (21.9)	9 (5.0)	23^d^ (28.8)	43^b^ (17.3)	365
2,1	168 (11.1)	106 (10.9)	37 (9.9)	16 (11.5)	3 (4.1)	21 (11.7)	3 (3.8)	23 (9.2)	377
2,2	406 (26.8)	266 (27.3)	102 (27.3)	40 (28.8)	13 (17.8)	64 (35.6)	9 (11.3)	57 (22.9)	957
2,3	177 (11.7)	128 (13.2)	54 (14.4)	16 (11.5)	4 (5.5)	10 (5.6)	31^d^ (38.8)	38 (15.3)	458
3,1	201 (13.2)	104 (10.7)	39 (10.4)	11 (7.9)	5 (6.8)	20 (11.1)	1 (1.3)	21 (8.4)	402
3,2	219 (14.4)	114 (11.7)	36 (9.6)	5 (3.6)	1 (1.4)	23 (12.8)	3 (3.8)	20 (8.0)	421
3,3	35 (2.3)	19 (2.0)	3 (0.8)	0 (0)	2 (2.7)	1 (0.6)	5 (6.3)	8 (3.2)	73
Total	1517	973	374	139	73	180	80	249	3585

OSA, obstructive sleep apnea; NSD, normal sleep duration; SSD, short sleep duration; Mod., Sev., V.Sev., are moderate, severe, and very severe OSA, respectively. Values are number of subjects with the type indicated; numbers in brackets indicate the percent of subjects in each clinical category with the type indicated. Differences between values in each category and the “No OSA/Insomnia” category were evaluated by the Chi-square test and their significance is indicated by superscripts in the column heading. ^a^*p* < .02; ^b^*p* < .0001; ^c^*p* < 1.E−5; ^d^*p* < 1.E−10; ^e^*p* < 1.E−25.

Table 3 shows the distribution of different ORP types in different clinical categories. All types were represented in subjects with “No OSA/Insomnia” with a contribution ranging from 2.0% (type “1,1”) to 26.8% (type “2,2”) of all subjects (Table 3). Distribution of patterns in mild OSA was not different from the “No OSA/Insomnia” category while distribution in moderate, severe, and very severe OSA was different from “No OSA/Insomnia” (Table 3). The difference from “No OSA/Insomnia” was exclusively because of progressively increasing representation of types “1,1”, “1,2”, and “1,3”. In very severe OSA, these types accounted for 61.7% of all subjects, compared to 20.3% in the “No OSA/Insomnia” category (*p* < .0001). The marked reduction in deep sleep (deciles 1 and 2) in severe and very severe OSA was independent of changes in proportion in full wakefulness (decile 10; Figure 6A).

Participants with insomnia-NSD had lower representation of epochs with excessive full wakefulness (Figure 6B) regardless of proportion in deep sleep. In contrast, distribution in insomnia-SSD was the opposite; all types with excessive full wakefulness (“1,3”, “2,3”, and “3,3”) were excessively represented (Table 3) accounting for 73.9% of participants with insomnia-SSD, by comparison to 22.6% of participants with “No OSA/Insomnia” (*p* < .0001). Importantly, while similar in proportion of epochs in full wakefulness, the two dominant types in insomnia SSD (“1,3” and “2,3”, Table 3) differed substantially from each other in the proportion of TRT in deep sleep (ORP < 0.5), which was very low in type “1,3” (5.8 ± 6.5%) and average in type “2,3” (16.6 ± 4.8%; *p* < .0001) (see also Figure 7).

### Sleepiness, and quality of life in different ORP architecture types

In unadjusted analyses, ORP types in the highest quartile of full wakefulness (“1,3”, “2,3”, and “3,3”) were associated with the three lowest ESS scores (7.2 ± 4.2, 7.4 ± 4.3, and 6.9 ± 4.3, respectively) while those in the lowest quartile of full wakefulness (“1,1”,” 2,1”, “3,1”) had three of the highest EES scores (7.9 ± 4.4, 8.0 ± 4.3, and 7.9 ± 4.3, respectively). Types in the lowest quartile of deep sleep (“1,1”, “1,2”, and “1,3”) had the lowest SF36-M scores while those with the highest amounts (“3,1”, “3,2”, and “3,3”) had three of the highest scores. Type “2,1” was associated with the most favorable SF36-P score and type “1,3” with the lowest (Table 4). Type “1.1” had the worst, or next to worst, scores in all three variables while type “3,1” had the best combined SF36 scores (4 of 18) with an average ESS.

**Table 4.** Sleepiness, and quality of life in different ORP architecture types

Type	n All/“No OSA/Insomnia”	Unadjusted (all subjects, *n* = 3585)			Adjusted for Age, gender, BMI, AHI, Insomnia. *n* = 3585			Adjusted for age, gender, BMI in “No OSA/Insomnia”. *n* = 1453		
		ESS (*p* < .004)	SF36(P) (p<0.0001)	SF36(M) (p<0.0004)	ESS(p=0.05)	SF36(P)(p=0.01)	SF36(M)(p=0.0001)	ESS(p=0.26)	SF36(P)(p=0.004)	SF36(M)(p=0.08)
1,1	107/29	8.2**[9]** (4.5)	46.1**[8]**(9.6)	50.9**[9]** (10.3)	−0.21 (0.42)	−0.44 (0.93)	−2.01 (1.03)	−1.25 (0.64)	0.13 (1.52)	−1.62 (1.52)
1,2	425/135	8.0**[7]** (4.6)	47.0**[5]**(9.9)	52.0**[8]**(9.2)	0.20 (0.23)	0.06 (0.47)	−1.31** (0.45)	−0.36 (0.33)	−0.49 (0.73)	−0.62 (0.76)
1,3	365/124	7.3**[2]** (4.3)	44.2**[9]**(10.9)	52.5 **[7] (9.1**)	−0.37 (0.22)	−1.96*** (0.58)	−1.17* (0.50)	−0.25 (0.37)	−2.86** (0.99)	−2.09* (0.91)
2,1	377/158	8.1**[8]** (4.4)	48.9**[1]**(9.1)	53.0**[6]**(8.6)	0.32 (0.23)	0.33 (0.48)	0.13 (0.45)	0.32 (0.34)	0.33 (0.69)	0.08 (0.69)
2,2	957/397	7.6**[4]** (4.4)	48.0**[4]**(9.3)	53.6**[5]**(8.0)	−0.00 (0.14)	0.50 (0.29)	0.37 (0.26)	0.26 (0.21)	0.72 (0.42)	0.02 (0.42)
2,3	458/170	7.0**[1]** (4.0)	46.8**[6]**(9.6)	53.8**[3]**(7.9)	−0.54** (0.19)	0.09 (0.43)	0.29 (0.38)	−0.54 (0.30)	0.53 (0.63)	0.25 (0.59)
3,1	402/195	7.8 [**6**] (4.3)	48.4**[2]**(9.7)	54.0**[2]**(7.4)	0.26 (0.22)	−0.14 (0.46)	1.04** (0.38)	0.18 (0.28)	−0.67 (0.63)	1.00* (0.52)
3,2	421/211	7.6**[5]** (4.2)	48.4**[3]**(9.3)	53.8**[4]**(7.9)	0.22 (0.21)	0.41 (0.42)	0.47 (0.40)	0.08 (0.30)	0.15 (0.55)	0.42 (0.59)
3,3	73/34	7.3**[3]** (4.3)	46.3**[7]**(10.5)	54.1**[1] (**9.3)	0.05 (.0.52)	−0.45 (1.17)	0.64 (1.09)	0.07 (0.78)	3.05* (1.22)	1.32 (1.40)

ESS, Epworth Sleepiness Scale; SF36(P), standardized score of SF-36 physical component Scale; SF36(M), standardized score of SF-36mental component Scale. For unadjusted variables, values are averages and SD (lower value in round brackets). Bold values in square brackets are the rank in the indicated variable and the ranks are from best to worst status. Values in the adjusted columns are mean (SEM). *p* values in the top row are from analysis of variance for differences between types in the relevant variable. Asterisks indicate significant association with ORP type after adjustment.

* *p* < .05, ** *p* < .01, *** *p* < .001.

In analyses adjusted for age, gender, BMI, AHI, and insomnia (middle section, Table 4) and including all subjects (*n* = 3585), the only type significantly associated with ESS was type “2,3” which had the lowest ESS. The three types with low amounts of deep sleep (“1,1”, “1,2”, “1,3”) continued to be associated with low SF36-M scores and type “1,3” was additionally negatively associated with SF36-P. Type “3,1” had significantly higher SF36-M (Table 4).

To assess whether these findings persist in Participants with no OSA or insomnia, adjusted analysis was repeated in subjects who have neither condition (*n* = 1453). Here, none of the types was associated with ESS, while type “1,3” continued to be negatively associated with SF36-P and SF36-M, and type “3,1” continued to be positively associated with SF36-M (Table 4, right columns). Type “3.3” was additionally positively associated with SF36-P.

### Summary of associations of ORP types with other ORP-derived variables

Table 5 shows the ranges of ORP_WAKE_, ORP_NREM_, ORP_TRT_, ORP-9, and cumulative sleep index (CSI) associated with the nine ORP types. ORP_WAKE_ ranged (10–90 percentile) 1.67–2.37 in the entire cohort while ORP_NREM_, ORP_TRT_ and ORP-9 ranged 0.44–1.49, 0.61–1.82, and 0.74–1.74, respectively. CSI ranged 321–920 ORP units * TRT. The ranges encountered in different ORP types were small relative to the overall ranges in the cohort (median (10%–90%) 31% (0.22–0.51). ORP_WAKE_ increased as percent in full wakefulness (2nd digit) increased while ORP_NREM_, ORP_TRT_, ORP-9 increased as the second digit increased and decreased as the first digit increased (Table 5). CSI decreased as the second digit increased and increased as the first digit increased. The net effect of these varied associations is that each ORP type was associated with a unique combination of the five ORP variables that incorporates differences between them in the various aspects of sleep depth, sleep propensity during stage wake, and CSI.

**Table 5.** Associations of ORP types with other ORP-derived variables

ORP Type	Range of values (10%–90%)				
	ORP_WAKE_	ORP_NREM_	ORP_TRT_	ORP-9	CSI
1,1	1.67–2.12 (Low)	0.88–1.20 (High)	1.02–1.34 (Average)	1.08–1.49 (High)	529–727 (average)
1,2	2.00–2.29 (Average)	0.91–1.35 (High)	1.17–1.53 (High)	1.16–1.65 (High)	454–641 (Low average)
1,3	2.16–2.37 (High)	0.94–1.49 (Very high)	1.36–1.82 (Very High)	1.17–1.74 (Very high)	321–544 (Low)
2,1	1.75–2.13 (Low)	0.68–0.91 (Average)	0.86–1.12 (average)	0.93–1.32 (Average)	630–791 (High average)
2,2	2.02–2.27 (Average)	0.70–0.99 (Average)	0.98–1.28 (Average)	0.97–1.39 (Average)	554–726 (Average)
2,3	2.17–2.36 (High)	0.69–1.04 (Average)	1.17–1.53 (High)	1.00–1.43 (Average)	454–640 (Low average)
3,1	1.68–2.10 (Low)	0.44–0.68 (Low)	0.61–0.89 (Low)	0.74–1.14 (Low)	719–920 (High)
3,2	1.99–2.23 (Average)	0.48–0.71 (Low)	0.78–1.06 (Low)	0.77–1.19 (Low)	657–843 (High)
3,3	2.17–2.35 (High)	0.49–0.70 (Low)	0.99–1.24 (Average)	0.79–1.20 (Average)	574–716 (Average)

ORP, odds ratio product; NREM, non-rapid eye movement sleep; TRT, total recording time; ORP-9, ORP in first 9 s after end of arousal; CSI, cumulative sleep index; OSA, obstructive sleep apnea; SSD, short sleep duration; ESS, Epworth sleepiness scale; SF36(P), standardized score of SF-36 physical component Scale; SF36(M), standardized score of SF-36 mental component Scale; Descriptors below the values refer to where the range is relative to the total range for the indicated variable. For ORP_WAKE_, low values indicate drowsy wakefulness, while low values for ORP_NREM_, ORP_TRT_ and ORP-9 indicate deep sleep, and faster sleep recovery after arousals; and vice versa.

## Discussion

We have introduced a new approach to describing sleep architecture based on the distribution of epochs with different sleep depth and wakefulness. This approach obviates many of the shortcomings of conventional methods of evaluating sleep depth/propensity. In addition, classification of ORP-based sleep architecture into distinct patterns (phenotypes) based on fractions of total recording in deep sleep and full wakefulness helps shed light on the underlying sleep pathophysiology and may help direct therapy. New insights resulting from this approach include: (1) Clearly abnormal ORP patterns are uncommon in individuals with mild/moderate OSA, but those with severe OSA demonstrated more drowsy wakefulness and transitional sleep and less deep sleep; (2) Contrary to individuals with insomnia-SSD, those with insomnia-NSD have significantly reduced amounts of full wakefulness; (3) There are two types of architecture in Participants with insomnia-SSD, one associated with poor sleep quality and quality of life, and the other with no such associations; (4) Architecture types that are associated with OSA, insomnia SSD, and reduced quality of life exist in subjects with neither OSA nor insomnia.

### Advantages of ORP-architecture

(1) ORP-architecture is based on objectively-determined EEG power in different frequencies relative to each other and is a metric that is directly related to sleep depth/arousability [4, 7]. In contrast, conventional architecture infers sleep depth from multiple EEG features that have less clear association to sleep depth and are subject to scorer differences (see Introduction and Supplementary Material). Figure 8 shows that the ORP values corresponding to deep sleep, transitional sleep, and full wakefulness do not correspond to their conventional counterparts.
(2) ORP-architecture describes sleep depth across the entire sleep and wake period whereas conventional architecture provides no information on variation in sleep depth during stage N2, which occupies the largest portion of recording time.
3) ORP-architecture distinguishes between epochs with full wakefulness (decile 10; Figure 1A, top) and those with drowsy wakefulness (ORP 1.75–2.25; Figure 1A, 2nd and 3rd panels). This distinction has important implications to the underlying pathophysiology in that a predominant increase in full wakefulness suggests a disorder with low sleep pressure in a significant fraction of the study (e.g., insomnia-SSD, circadian misalignment…etc.), while a predominant increase in drowsy wakefulness suggests a disorder that interferes with sleep progression (e.g., OSA, other sources of arousal stimuli) and results in high sleep pressure during wake time.

Comparing results of conventional metrics (Table 2) and ORP-metrics (Table 3) illustrates some of the advantages of the proposed approach. First, other than increased wake time, implicit in insomnia-SSD’s definition, conventional metrics were similar between insomnia-NSD and insomnia-SSD, or between either and sleep depth in participants without insomnia (Table 2). In contrast, ORP patterns identified several differences: (a) As opposed to participants with insomnia-SSD, those with insomnia-NSD had less full wakefulness than individuals with no insomnia (Figure 6B); (b) In insomnia-NSD, the predominant ORP patterns were not different from those in individuals without insomnia but were substantially different than patterns observed in individuals with insomnia-SSD (Table 3); (c) there were two predominant patterns in insomnia-SSD, one with very little deep sleep (pattern “1,3”) and the other with normal amounts of deep sleep (as defined by pattern “2,3”), that have different associations with health outcomes (Table 4). Thus, it is possible that individuals with insomnia-SSD with type 1,3 are those who are most likely to experience the poor health outcomes that have more generally been attributed to subjects with insomnia-SSD [19–22].

Second, while sleep disorders are associated with statistically significant associations with conventional metrics in large cohorts [23], the range of each conventional metric is so wide in the general population that it is not possible to ascertain whether a certain metric is abnormal in a given individual (Table 2). In contrast, the different ORP types are associated with narrow ranges in metrics that describe sleep propensity (ORP_WAKE_) and sleep depth (Table 5).

Third, with conventional metrics severity of OSA is evaluated from the AHI and the closely related arousal index. The present approach has identified several ORP patterns within each AHI category (Table 3). Some of these seem to have no association with poor health outcomes (Table 4). In addition, the three dominant patterns in OSA differ from each other in the amount of time in full wakefulness, with one having very little (“1,1”) while the other (“1,3”) is associated with excessive amounts, thereby reflecting different underlying pathophysiology (high vs. low sleep pressure). Furthermore, the current results show that impairment of sleep is not linearly related to AHI. Thus, mild and moderate OSA are associated with very little changes in sleep depth (Table 3) while frequency of impaired sleep depth (% of subjects with first digit of 1) increases dramatically in more severe cases. Finally, even in very severe OSA (AHI >50 h) 38% of Participants have normal sleep depth and ORP patterns that are unassociated with poor quality of life or excessive sleepiness. These observations may explain the three clinical subtypes described in participants with OSA at similar AHI, namely sleepy type, sleep disturbed type and minimally symptomatic type [24–26].

### Association of demographics and BMI with ORP-defined sleep architecture

The current results are in general agreement with well-established observations that sleep generally deteriorates with advanced age both in duration (TST, SE) and in depth (proportions of N1 and N3) [27]. ORP analysis additionally revealed that age-related changes in sleep depth are not limited to an increase in light sleep (i.e. N1) or a decrease in deep sleep (i.e. N3) but represent a general shift to lighter sleep across all epochs (Figure 4A). Furthermore, the increase in wake time with age was mostly due to increased time in full wakefulness (decile 10, Figure 4). This suggests that increased wake time with age is due to lower sleep drive rather than to disorders disrupting sleep, which would have been associated with increased drowsy sleep.

This study is also the first to report cumulative sleep amount (CSI) and this showed a 33% reduction from young adults to older adults (Figure 4A, inset). This much lower amount along with the associated increase in time in full wakefulness, suggests that age-related changes in sleep architecture may, at least in part, be related to decrease in sleep need as we age.

Prior studies have reported that women have lower N1 and higher N3 sleep than men [23]. In contrast, we found only very small differences between men and women with “No OSA/Insomnia” in ORP architecture (Figure 4B, Supplementary Table S1) and CSI (inset, Figure 4B), suggesting differences in epoch-specific classification of sleep stages vs. ORP depth distribution.

Rao et al. found that N3 duration was negatively associated with BMI [28]. We did not find an association between BMI and percent of epochs with ORP-defined deep sleep (ORP < 0.50; Figure 4C). The discrepancy may be related to the fact that N3 is identified by slow, large delta waves (≤2.0 Hz and amplitude >75 μV) [9], whereas ORP-determined deep sleep is sensitive to the faster component of delta power (2.5–5.0 Hz) [4]. Hence, these two metrics do not measure the same thing (Figure 8B). The association of stage N3 and BMI may therefore specifically reflect an association of BMI with the amount of large delta waves. We found earlier that once these large delta waves appear (generally when delta power is >300 μV^2^), sleep depth no longer increases as their frequency or size increases [7].

### Rationale for using ORP types

The division of the ORP range into deciles is consistent with the original description of ORP distribution during sleep studies [4]. It is also a reasonable compromise between having fewer bins with each bin having a wide range of sleep depth, and having too many bins that include very small ranges of little clinical significance while being too cumbersome to describe. Furthermore, given that epochs with ORP > 1.75 are generally scored wake [4], division by deciles provides three values in the wake range (8th, 9th, and 10th deciles), which permit distinguishing full from drowsy wakefulness. Nonetheless, use of deciles resulted in distribution patterns that are difficult to characterize by simple mathematical functions (e.g. Figures 2 and 7) that can be used to characterize sleep and wakefulness in easy to use terms. Hence, we relied on functional criteria to break the patterns into a reasonable number that define features of clinical significance.

The use of a two-digit descriptor of the different patterns is also more useable and instructive than using a complex function with various constants that have no functional counterparts. With the two-digit descriptor the user can easily conclude that pattern “1,1” means little time in deep sleep and little time in full wakefulness, and so on.

Our principal assumption in classifying ORP types is that time spent in full wakefulness reflects time spent with low sleep pressure, and vice versa. This assumption was validated in this study in several ways: (1) Time spent in full wakefulness decreases in the presence of sleep loss and increases as sleep pressure wanes (Figure 5); (2) Time in full wakefulness is associated with lower ESS (Table 1); (3) ORP types associated with very little time in full wakefulness (“1,1”, “2,1”, and “3,1”) are associated with the highest ESS scores in unadjusted analyses, and vice versa (Table 4). The other main assumption is that low levels of deep sleep associated with low levels of full wakefulness represent different pathophysiology than low levels of deep sleep with high levels of wakefulness, and vice versa. This was confirmed previously [5] and also here. Thus, type “1,1” is most prevalent in OSA where sleep fragmentation is peripherally mediated while it was not seen in individuals with insomnia-SSD where the light sleep is of central origin (Table 3).

The statistical approach used to determine cut-off levels for the three ranks in each of the two digits (i.e. which quartile each value lies) is also standard, free of bias as it was decided on a priori, and based on ranges found in a large community based cohort that included many participants with OSA and insomnia. However, further research is needed to address how generalizable these cutoffs are to other samples.

It may be argued that our use of the extremes of the ORP range to define the types ignores the results between the two extremes. However, we found that each type, so selected, was associated with a narrow range in the other relevant ORP metrics (Table 5) and specific health outcomes (Table 4). Thus, what happens between the two extremes is reflected at the two extremes.

### New insights with potential clinical implications

Table 6 is a grand summary of the main characteristics of each ORP architecture type (left column), what each pattern physiologically could represent (second column), associations with demographics, clinical phenotypes, and health outcomes (third column), and potential clinical implications that can be pursued in clinical studies (fourth column). All comments are based on time in bed >7 h. In addition to the observations in the table, the following general observations may be clinically relevant:

(1) Patterns “1,3” and “2,3” are rare in young adults (Figure 9). Such patterns in young adults might indicate an insomnia disorder. On the other hand, type “3,1” in an individual of advanced age might suggest sleep deprivation, as this type is rare in older people (Figure 9).
(2) Of the nine ORP types only three, “1,1”, “1,2”, and “1,3”, are predominant in sleep disorders (Table 3) and are associated with participant-centered outcomes of sleepiness and reduced health-related quality of life (Table 4). The presence of these three types in someone with sleep complaints with no obvious sleep pathology on PSG may warrant further evaluation for an underlying sleep disorder.
(3) The occurrence of type “1,3” in someone with severe OSA but no insomnia may reflect a coexistent hyperarousal state, which may be considered during therapy (e.g. use of cognitive behavioral therapy [29, 30] or sedatives along with CPAP).

**Table 6:** Summary of characteristics of different ORP types and their suggested interpretation and potential clinical implications

ORP-Architecture Type*	Suggested Underlying Physiological Mechanism	Clinical Associations	Suggested Clinical Interpretation
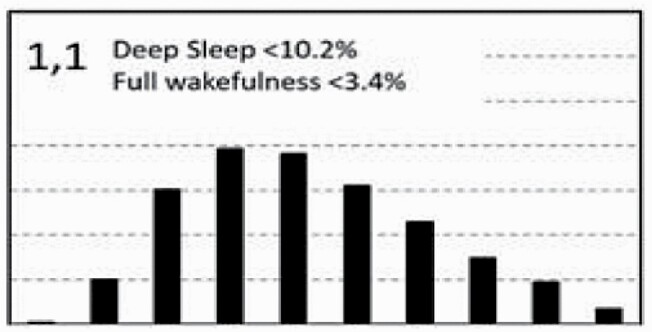	Little deep sleep suggests either low sleep drive or sleep-fragmenting disorder (OSA, other sources). Little full wakefulness and low ORP_WAKE_ (Table 5) favor high, not low, sleep drive. Conclusion: Likely sleep fragmenting disorder ® high sleep pressure.	Rare at all ages in the general community (Figure 9). Frequency increases with OSA severity (Table 3). Associated with poor sleep quality (Table 5) and low QOL in unadjusted analysis (Table 4).	Suggestive of a severe sleep-fragmenting disorder. Warrants investigation of cause. Cause may be evident in PSG (OSA, PLMs) or arousal stimuli may originate from other sources (pain, itching, etc.). If associated with OSA sleep is likely to improve with R_X_.
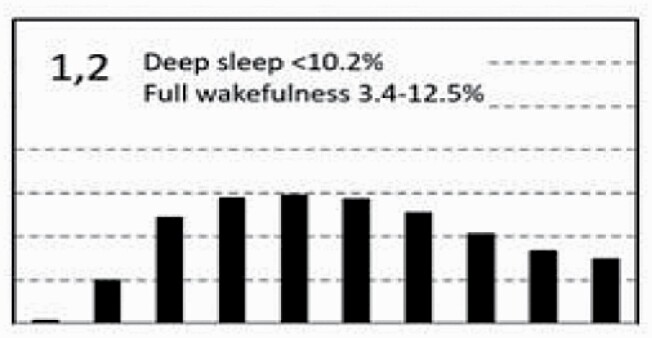	Little deep sleep suggests low sleep drive or sleep-fragmenting disorder. But average full wakefulness and normal ORP_WAKE_ (Table 5) argue against low sleep drive. Conclusion: Likely sleep-fragmenting disorder.	Not uncommon in people with no OSA/insomnia (Table 3). Frequency increases with age (Figure 9) and OSA severity (Table 3). Associated with poor sleep quality (Table 5) and reduced QOL (Table 4).	Same as type 1,1, but less likely to be sleepy. May represent a mild form of type 1,3 (low sleep drive) particularly in the absence of an organic sleep-fragmenting disorder.
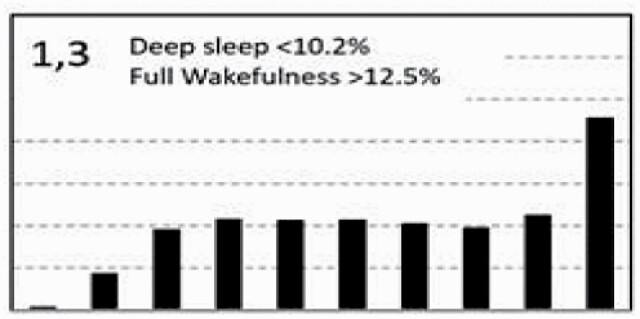	Little deep sleep suggests either low sleep drive or sleep-fragmenting disorder (OSA, other sources). But increased amount of full wakefulness and high ORPWAKE and ORP-9 (Table 5) strongly suggest low sleep drive.	Rare in young subjects but frequency increases with age (Figure 9) and markedly in very severe OSA, insomnia SSD and COMISA (Table 3). Associated with very poor sleep quality (Table 5) and reduced QOL (Table 4).	Normal in old people, particularly if asymptomatic. Occurrence in younger people or symptomatic older people is suggests a hyperarousal state. Frequently associated with severe OSA where concurrent Rx of insomnia may be considered.
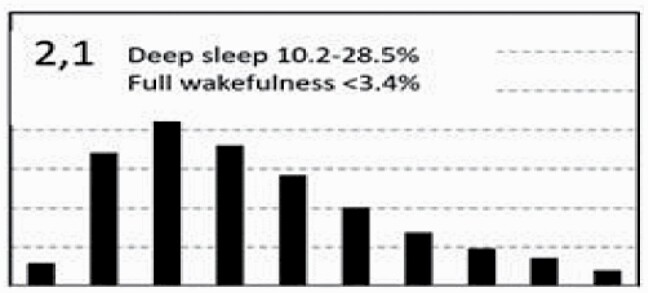	Average amounts of deep sleep and sleep depth (Table 5). Little time in full wakefulness and low ORP_WAKE_ (Table 5) suggest insufficient sleep.	Average frequency with no tendency to increase in OSA or insomnia (Table 3). No association with reduced QOL (Table 4).	Likely normal but may benefit from increasing time in bed if excessively sleepy.
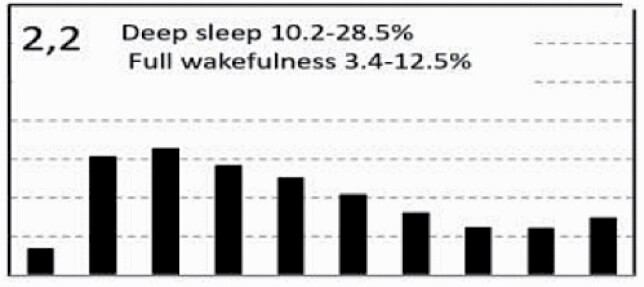	Average deep sleep and normal sleep depth (Table 5) suggest normal sleep. Presence of moderate amount of full wakefulness suggests adequate restorative function.	Most frequent pattern in subjects without OSA or insomnia. Tendency to be lower in severe OSA and insomnia SSD (Table 3). No associated adverse health outcomes (Table 4).	Normal sleep. Symptoms, if any, are likely not related to poor sleep.
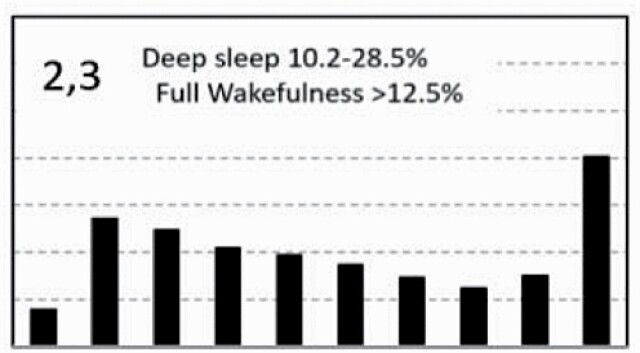	Average deep sleep and sleep depth (Table 5) suggest adequate sleep quality. Excessive amount of full wakefulness despite adequate sleep quality suggests reduced sleep need or circadian misalignment.	Frequency increases markedly in old individuals (Figure 9) and dramatically in insomnia-SSD (Table 3). Associated with normal QOL on average (Table 4). Normal in the elderly but circadian misalignment possible if symptoms present.	Suggests decreased sleep need (short sleeper). Normal in the elderly if asymptomatic. Daytime symptoms suggest circadian misalignment or lifestyle issues. May be a common, less malignant form of insomnia-SSD.
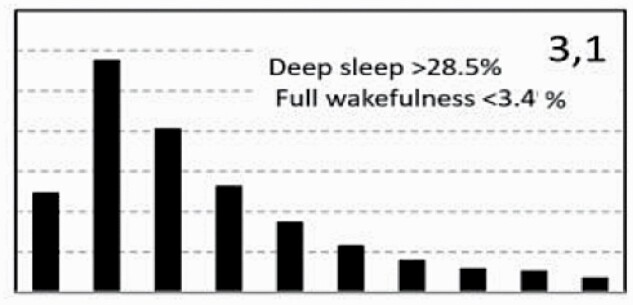	Excessive amount of deep sleep suggests prior sleep deprivation (Figure 5A) or chronic insufficient sleep. Little time in full wakefulness and low ORP_WAKE_ (Table 5) suggest that sleep was not completely restorative.	Most frequent type in healthy young adults (Figure 9). Rare in older subjects (Figure 9). Tendency to lower frequency in severe OSA and insomnia-SSD. Associated with above average QOL (Table 4).	Normal in young adults. Presence in older adults or symptomatic young adults suggests prior sleep deprivation (Figure 5A). In absence of recent sleep loss, excessive sleep need (long sleeper, central hypersomnolence) is suggested.
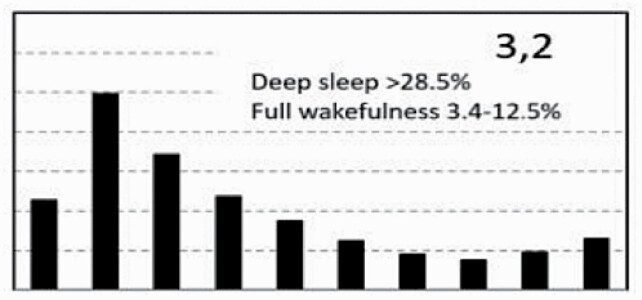	Excessive amounts of deep sleep suggest prior sleep deprivation (Figure 5A) but moderate amount of full wakefulness and average ORP_WAKE_ argue against sleep deprivation.	No association with age (Figure 9) or gender (Supplementary Table S1). Tendency to be less frequent in severe OSA and insomnia SSD (Table 3). No associated adverse health outcomes (Table 4).	Normal sleep. Symptoms, if any, are likely not related to poor sleep.
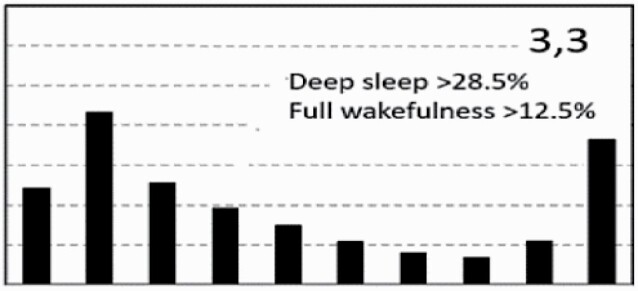	Combination of increased deep sleep and excessive amount of full wakefulness suggests short sleeper or circadian misalignment (see type 2,3, above).	Rare in all demographics (Figure 9 and Supplementary Table S1) with no association with OSA or insomnia (table 3). Not associated with adverse health outcomes (Table 4).	Suggests decreased sleep need if asymptomatic. Daytime symptoms suggest circadian misalignment or lifestyle issues.

* Deep sleep refers to epochs with ORP < 0.5. Full wakefulness refers to epochs with ORP > 2.25. ORP, odds ratio product; OSA, obstructive sleep apnea; PLMs, periodic limb movement. QOL, quality of life. SSD, short sleep duration.

### Limitations

(1) The reference for the age-related changes in ORP architecture (Figure 4A) was the youngest group and this group was from a different cohort than the three other age groups. This limitation is mitigated by several considerations: (a) As in the twin cohort, the SHHS subjects used for age-related changes were free of OSA and insomnia. (b) Progression of ORP changes with age (Figure 4A) does not show any discontinuity between the <40 group and the three subsequent groups.
(2) We acknowledge the multiple comparisons and large sample size may have identified some differences that were statistically but not clinically significant.
(3) Insomnia was based on a rating scale, as used in other epidemiological studies, and not on clinical assessment.
(4) Studies of short recording durations were not included. Although it is likely the majority of these were due to low battery life, it is possible that individuals with the shortest sleep times may have been excluded.

## Conclusions

We have described a new ORP-based approach to evaluating and describing sleep architecture, providing a comprehensive and unique description of the quantity and intensity of wake suppression during the study. The ORP based metrics varied with self-reported sleepiness and age, providing evidence for convergent validity, while providing novel associations with several sleep phenotypes. In addition, it is expressed in easily recognizable patterns that shed light on the underlying mechanism(s) of sleep disorders, and may provide new tools for diagnosing, classifying, and managing sleep disorders.

## Supplementary Material

zsac059_suppl_Supplementary_Material

## Data Availability

The data underlying this article will be shared on reasonable request to the corresponding author.
