# Supplementary material for: Sleep architecture based on sleep depth and propensity: patterns in different demographics and sleep disorders and association with health outcomes
Source: Sleep. 2022 Mar 10;45(6):zsac059. doi: 10.1093/sleep/zsac059 (PMC9195236; doi:10.1093/sleep/zsac059)
Supplement: zsac059_suppl_Supplementary_Material [file zsac059_suppl_supplementary_material.docx]

Online Supplement

Sleep Architecture Based on Sleep Depth and Propensity: Patterns in Different Demographics and Sleep Disorders and Association with Health Outcomes.

Magdy Younes, Bethany Gerardy, Allan I. Pack, Samuel T. Kuna, Cecilia Castro-Diehl, Susan Redline

***Shortcomings of Conventional Architecture Metrics***

1) Sleep efficiency simply reflects the percent of recording time spent in epochs that meet the arbitrary definition of the wake state.^(1)^ Yet the EEG in such epochs can range from patterns that are not very dissimilar from stage 1 NREM sleep, including periods of microsleep, to patterns of full wakefulness with continuous intense high frequency activity.^(2)^ Recent evidence suggests that the EEG pattern in epochs staged wake is sensitive to sleep loss and may help identify patients with increased sleep pressure.^(3)^

2) The AASM requirement to change NREM sleep stage to NREM1 following an arousal until a spindle or k complex occurs^(1)^ all but invalidates NREM1(%) as an independent measure of sleep depth. Because of this rule NREM1% adds little beyond the arousal index; there is an excellent correlation between NREM1% and the arousal index.^(4)^ Furthermore, the extent to which arousals increase NREM1% depends on how often spindles occur, which ranges from 1 every 4 minutes to 4 per minute.^(5)^ Thus, each arousal may add anywhere from 0 (when spindles are very frequent) to 4 minutes to NREM1 time. In addition, frequency of spindle occurrence (spindle density) has little to do with sleep depth. In fact, spindle density is inversely proportional to sleep depth.^(6,7)^

3) The ability of technologists to estimate the total duration of qualifying delta waves and, by extension, stage NREM3 is extremely limited.^(8)^ Furthermore, we have recently shown that visually-scored NREM3 time is strongly affected by overall EEG amplitude (Normalized EEG power), with subjects having high EEG power across all frequency ranges receiving more scored NREM3 time than those with low EEG power.^(2)^

4) Most importantly, NREM1 and NREM3 typically occupy small fractions of sleep time that represent the extremes of sleep depth. Meanwhile, most of the changes in sleep depth occur within stage NREM2,^(2,4)^ and this stage occupies the largest fraction of sleep time. Conventional metrics do not give any indication of sleep depth within stage NREM2.

5) There is more than one conventional metric of sleep depth, and these often do not change harmoniously change (e.g., arousal index improves while NREM3% decreases),^(4)^ making it difficult to determine whether sleep depth improved.

6) The arousal index is simply a count of sporadic events that does not consider their duration or intensity. However, arousal duration ranges from 3 to 15 seconds,^(1)^ and their intensity can range from barely discernible to very intense.^(9)^ Furthermore, the after-effect of arousals on sleep quality/depth depends on the speed of sleep depth recovery following arousals; arousals impact overall sleep depth more severely in those in whom sleep depth returns very slowly to pre-arousal levels beyond the arousal.^(10)^

**Method of Determining ORP:**

As described previously,^(11)^ ORP is calculated by applying fast Fourier analysis to 3-second consecutive non-overlapping segments of the EEG (central or frontal derivations) throughout the entire polysomnogram (PSG). The entire process to be described here is executed automatically and consumes less than one minute for an entire 8-hour PSG. Total power in 4 frequency ranges is calculated in each three second segment (0.33-2.33 Hz, 2.67-6.33 Hz, 7.0-14.0 Hz, and 14.3-35.0 Hz). The power in each frequency range is assigned a rank (0 to 9) by reference to a dataset containing the power in said frequency measured in >400,000 artifact-free 3-second epochs obtained from a wide range of clinical PSGs. The four ranks are concatenated in a 4-digit number (from left to right, lowest to highest frequency rank), thereby generating 10,000 unique combinations (Bin numbers). Thus, the 4-digit number 9138 describes an EEG segment having very high powers in the slowest and fastest frequencies, very little power in the 2^nd^ frequency and moderate power in the 3^rd^ frequency.

The probability of each Bin number occurring in 30-second epochs scored by expert technologists as wake, or during arousal within epochs scored sleep, is determined by reference to another look-up table. For each Bin number the look-up table indicates; a) the total number of epochs with each Bin number found in the entire dataset referred to above, b) the number of times it was found during wake epochs or within arousals and, c) b*100/a, representing the desired probability. The probability range (0-100%) is normalized by dividing by 40 (% of epochs scored wake in the reference files) thereby generating an ORP range of 0.0-2.5, with an ORP of zero indicating that the pattern never occurs during wake epochs or arousals and 2.5 indicating a pattern that is never seen during sleep.

REFERENCES

1. Berry RB, Brooks R, Gamaldo CE, Harding SM, Marcus CL and Vaughn BV for the American Academy of Sleep Medicine. The AASM Manual for the Scoring of Sleep and Associated Events: Rules, Terminology and Technical Specifications, Version 2.0. www.aasmnet.org, Darien, Illinois: American Academy of Sleep Medicine, 2012.
2. Younes M, Azarbarzin A, Reid M, Mazzotti DR, Redline S. Characteristics and Reproducibility of Novel Sleep EEG Biomarkers and their Variation with Sleep Apnea and Insomnia in a Large Community-Based Cohort. Sleep. In Press.
3. Younes M, Schweitzer P, Griffin K, Balshaw R, Walsh J. Comparing two measures of sleep depth/intensity. Sleep. 2020 Dec 14;43(12): zsaa127.
4. Qanash S, Giannouli E, Younes M. Assessment of intervention-related changes in non-rapid-eye-movement sleep depth: importance of sleep depth changes within stage 2. Sleep Med. 2017 Dec;40:84-93.
5. Younes M. The case for using digital EEG analysis in clinical sleep medicine. Sleep Sci Practice. Sleep Science and Practice. 2017-1:2.
6. Curcio G, Ferrara M, Pellicciari MC, Cristiani R, De Gennaro L. Effect of total sleep deprivation on the landmarks of stage 2 sleep. Clin Neurophysiol. 2003;114(12):2279-85.
7. De Gennaro L, Ferrara M. Sleep spindles: an overview. Sleep Med Rev. 2003 Oct;7(5):423-40.
8. Younes M, Kuna ST, Pack AI, Walsh JK, Kushida CA, Staley B, Pien GW. Reliability of the American Academy of Sleep Medicine Rules for Assessing Sleep Depth in Clinical Practice. J Clin Sleep Med. 2018 Feb 15;14(2):205-213.
9. Azarbarzin A, Ostrowski M, Hanly P, Younes M. Relationship between arousal intensity and heart rate response to arousal. Sleep. 2014 Apr 1;37(4):645-53.
10. Younes M, Hanly PJ. Immediate post-arousal sleep dynamics: an important determinant of sleep stability in obstructive sleep apnea. J Appl Physiol. 2016 Apr 1;120(7):801-8.
11. Younes M, Ostrowski M, Soiferman M, Younes H, Younes M, Raneri J, Hanly P. Odds ratio product of sleep EEG as a continuous measure of sleep state. Sleep. 2015;38(4):641-54.

Table S1.

| TABLE S1 | | | | | | | | | | | | |
| --- | --- | --- | --- | --- | --- | --- | --- | --- | --- | --- | --- | --- |
| Distribution of ORP Patterns by Gender and Age | | | | | | | | | | | | |
| ORP Pattern | AGE Males | | | | Total male | AGE Females | | | | Total Female | % Total Males | % Total Females |
|  | 19-39 | 40-54 | 55-69 | 70-90 |  | 19-39 | 40-54 | 55-69 | 70-90 |  |  |  |
| 1,1 | 1 | 3 | 4 | 3 | 11 | 7 | 8 | 5 | 7 | 27 | 1.9 | 2.4 |
| 1,2 | 1 | 16 | 30 | 12 | 59 | 7 | 23 | 41 | 25 | 96 | 10.3 | 8.6 |
| 1,3 | 0 | 5 | 22 | 22 | 49 | 1 | 9 | 42 | 32 | 84 | 8.6 | 7.5 |
| 2,1 | 10 | 32 | 30 | 10 | 82 | 27 | 55 | 28 | 12 | 122 | 14.3 | 10.9 |
| 2,2 | 17 | 43 | 72 | 19 | 151 | 31 | 105 | 117 | 53 | 306 | 26.4 | 27.4 |
| 2,3 | 0 | 9 | 21 | 26 | 56 | 4 | 24 | 49 | 48 | 125 | 9.8 | 11.2 |
| 3,1 | 18 | 27 | 25 | 6 | 76 | 34 | 76 | 54 | 13 | 177 | 13.3 | 15.9 |
| 3,2 | 4 | 26 | 37 | 13 | 80 | 10 | 47 | 76 | 19 | 152 | 14.0 | 13.6 |
| 3,3 | 0 | 2 | 4 | 2 | 8 | 0 | 6 | 10 | 11 | 27 | 1.4 | 2.4 |
| Total | 51* | 163 | 245 | 113 | 572 | 121* | 353 | 422 | 220 | 1116 | 100.0 | 100.0 |
| ORP, Odds ratio product. No significant difference between males and females at any age group or when all ages | | | | | | | | | | | | |
| are pooled (p>0.25 for all comparisons by the Chi-square test). *Gender not available in two twin pairs | | | | | | | | | | | | |


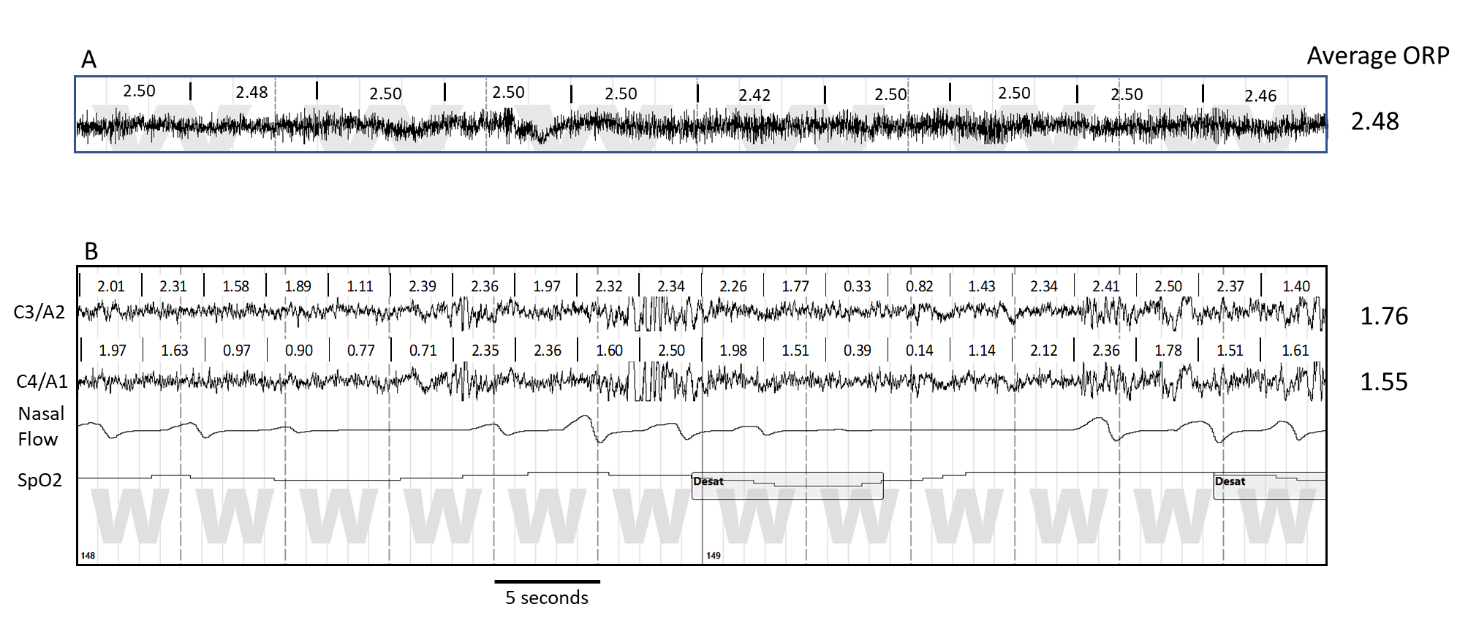


Figure S1: A) 30-second epoch with full wakefulness. Note that odds ratio product (ORP) is close to the maximum value of 2.5 in each 3-second epoch and in the average of the 10 values. B) A pattern frequently seen in severe OSA. Two 30-second epochs also scored wake but with low odds-ratio product (ORP). Numbers above the tracings are 3-second ORP values. Note that when ORP transiently decreases, indicating micro-sleep, the patient develops an apnea which interrupts progression to deeper sleep.
